# A Growing Link between Circadian Rhythms, Type 2 Diabetes Mellitus and Alzheimer’s Disease

**DOI:** 10.3390/ijms23010504

**Published:** 2022-01-03

**Authors:** Xuemin Peng, Rongping Fan, Lei Xie, Xiaoli Shi, Kun Dong, Shujun Zhang, Jing Tao, Weijie Xu, Delin Ma, Juan Chen, Yan Yang

**Affiliations:** 1 Department of Endocrinology, Tongji Hospital, Tongji Medical College, Huazhong University of Science and Technology, Wuhan 430030, China; m201976046@hust.edu.cn (X.P.); m202076148@hust.edu.cn (R.F.); m202176128@hust.edu.cn (L.X.); shixiaoli@tjh.tjmu.edu.cn (X.S.); kundong@tjh.tjmu.edu.cn (K.D.); sjzz899@tjh.tjmu.edu.cn (S.Z.); tjskc@tjh.tjmu.edu.cn (J.T.); wuweijie@tjh.tjmu.edu.cn (W.X.); dma@hust.edu.cn (D.M.); 2 Branch of National Clinical Research Center for Metabolic Diseases, Wuhan 430030, China; 3 Department of Neurosurgery, Tongji Hospital, Tongji Medical College, Huazhong University of Science and Technology, Wuhan 430030, China; jchen@tjh.tjmu.edu.cn

**Keywords:** type 2 diabetes mellitus, Alzheimer’s disease, circadian rhythms, therapy

## Abstract

Type 2 diabetes mellitus (T2DM) patients are at a higher risk of developing Alzheimer’s disease (AD). Mounting evidence suggests the emerging important role of circadian rhythms in many diseases. Circadian rhythm disruption is considered to contribute to both T2DM and AD. Here, we review the relationship among circadian rhythm disruption, T2DM and AD, and suggest that the occurrence and progression of T2DM and AD may in part be associated with circadian disruption. Then, we summarize the promising therapeutic strategies targeting circadian dysfunction for T2DM and AD, including pharmacological treatment such as melatonin, orexin, and circadian molecules, as well as non-pharmacological treatments like light therapy, feeding behavior, and exercise.

## 1. Introduction

There are 40 million people suffering from dementia all over the world, which is estimated to double every 20 years until at least 2050, adding a tremendous burden to the economy and health worldwide [1]. The most common cause of dementia is Alzheimer’s disease (AD), which has presented one of the greatest healthcare challenges of the 20th and 21st centuries. The main characteristics of AD are intracellular neurofibrillary tangles (NFTs) caused by tau hyperphosphorylation and the accumulation of amyloid plaques produced by amyloid β (Aβ) [2].

Type 2 diabetes mellitus (T2DM) is the most common metabolic disease, characterized by hyperglycemia and insulin resistance, combined with relative insulin deficiency. The number of T2DM patients is increasing worldwide and is estimated to rise to 642 million by 2040, causing severe public health challenges [3]. Previous studies observed brain AD pathology in over 40% of T2DM patients at the time of death [4]. One meta-analysis in 2017 included over 17 original studies with more than 1.7 million participants and estimated that the relative risk of AD in diabetic patients was 1.36 (95% CI 1.18–1.53) in western populations and 1.62 (95% CI 1.49–1.75) in eastern populations [5]. Ninomiya found that patients with diabetes had a pooled hazard ratio (HR) for Alzheimer’s disease (HR = 1.6 (95% CI 1.4–1.8)) [6]. These epidemiological studies have suggested that T2DM patients are at higher risk of developing AD, indicating a strong association between these two diseases. The factors of insulin resistance, inflammation, oxidative stress and glycogen synthase kinase 3β signaling may be involved in the complex association between T2DM and AD [7]. Although many different hypotheses have been proposed, the treatments targeting AD aresymptomatic for the most part [8]. 

Mounting evidence indicated that circadian disruption may be closely associated with both T2DM and AD [8,9]. Therefore, innovative strategies involving circadian rhythms seem attractive in treating T2DM and AD. In this review, we will introduce the concept of circadian rhythms and the association between circadian disruption with T2DM and AD, through summarizing the evidence from both human and animal research. Importantly, the attractive strategies involving circadian rhythms in the therapy of T2DM and AD will also be discussed in this review.

## 2. Circadian Rhythms

**Figure 1:**
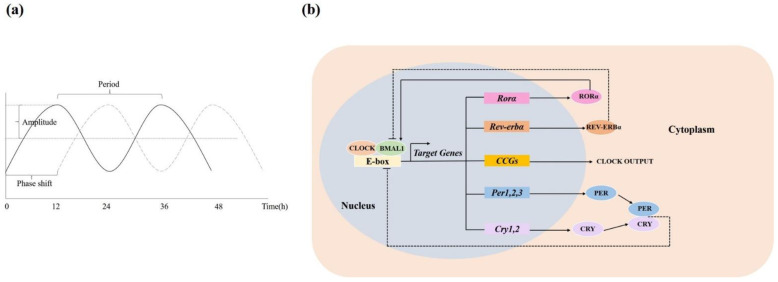
Molecular mechanism of the circadian clock. (**a**) The three parameters (amplitude, phase and period) of circadian rhythmicity. (**b**) In the TTFL, the positive transcriptional limb CLOCK and BMAL1 drive the expression of negative feedback repressors (PER, CRY), which subsequently suppress the expression of the positive limb. Additional feedback loops include REV-ERBα and RORα. REV-ERBα stimulates the gene expression of Bmal1, while RORα inhibits Bmal1 gene expression. CLOCK and BMAL1 also regulate a variety of clock-controlled genes (Ccg) that mediate the downstream circadian clock output. (In Figure 1b, straight lines: stimulation; dashed lines: inhibition.).

In order to adapt to environmental changes, all animals and plants exhibit an approximately 24-h cycle to synchronize biological function, which is known as the circadian rhythm [10]. In humans, the typical examples of circadian rhythms include sleep-wake cycles, fluctuations of blood pressure and core body temperature, and the release of various hormones, such as melatonin, cortisol, etc. [11]. The circadian rhythmicity is typically defined by three parameters: amplitude, phase and period. Amplitude represents the magnitude of cycles, which reflects the strength of the rhythm. Phase represents the timing of a reference point relative to a fixed point. Period is defined as the time interval between two recurring waves within a rhythm (Figure 1a) [12].

In mammals, circadian rhythms are self-sustained and are generated by a hierarchical timekeeping system containing the master circadian clock, which is situated in the suprachiasmatic nucleus (SCN) of the hypothalamus and peripheral clocks in most organs. The retinohypothalamic tract receives light signals and delivers them to the SCN, to synchronize the endogenous “clockwork”. In turn, the SCN communicates with a variety of peripheral clocks in the brain regions and most organs like the heart, liver, muscle, pancreas, and adrenal system through synaptic and diffusible signals. Therefore, the SCN, as the central clock, receives photic inputs such as light signals and synchronizes peripheral clocks throughout almost all cell types and organs [10]. In addition, the non-photic signals, including food, temperature, exercise, and social activities, could also be transmitted to the SCN as well as the peripheral clocks, and corresponding adaptive changes may be produced [13].

At the molecular level, the circadian clock is composed of a group of proteins, forming a cell-autonomous transcriptional-translational feedback loop (TTFL) that mediates daily oscillations in gene expression [14]. In this TTFL, the positive transcriptional limb consists of “circadian locomotor output cycles kaput” (CLOCK) and the “brain and muscle arnt-like protein-1” (BMAL1), driving the expression of their negative feedback repressors such as Period (PER1, PER2 and PER3) and cryptochrome (CRY1/CRY2) genes, which subsequently suppress the expression of the positive limb [14]. There are also additional feedback loops involving REV-ERBα (reverse erythroblastosis virus α) and RORα (retinoid-related orphan receptor-α), which also regulate the gene expression of *Bmal1*. In addition, the “clock” genes also regulate a variety of downstream target genes called clock-controlled genes (*Ccg*), which are involved in energy and metabolism, immune responses, oxidative processes, and other functions (Figure 1b) [15,16]. Therefore, it is the TTFL in the SCN and peripheral clocks that maintains 24-h rhythms in gene expressions, which are necessary for physiological and behavioral rhythmicity. When the circadian rhythms are disrupted (such as altered light/dark (LD) cycles, jet lag, shift work, or chronic sleep deprivation), there will be a greater occurrence of many diseases like T2DM and AD [17,18].

## 3. T2DM and Circadian Disruption 

In recent years, growing evidence has suggested a close relationship between circadian rhythms and T2DM. The glucose metabolism displays circadian cycles, disruption of which leads to the occurrence and development of diabetes [19,20,21].

### 3.1. The Diurnal Rhythm of Glucose Metabolism

As early as the 1960s and 1970s, several studies demonstrated the presence of a diurnal rhythm of blood glucose in oral glucose tolerance, which was lower in the afternoon and evening than in the morning [22,23,24,25]. Subsequently, mounting evidence further confirmed the circadian rhythm of blood glucose using intravenous glucose, insulin tolerance tests, glucose infusions, identical meal tests, and enteral nutrition [26,27,28]. These diurnal oscillations in glucose tolerance may be partially due to the diurnal rhythms of β-cell responsiveness, secretion and the clearance of insulin because blood insulin levels and insulin sensibility also follow circadian rhythms [23,29,30]. In addition, glucagon and glucagon-like peptide-1, which are both necessary for the regulation of blood glucose, are shown to vary according to circadian rhythms [31,32].

### 3.2. T2DM Animals and Patients Show Circadian Disruption

Of the two conditions, T2DM is more prone to circadian disruption. A substantial number of studies have demonstrated that diabetes is always accompanied by the disruptive rhythms of glucose metabolism [22,23,26,33]. As early as the 1960s, Jarrett et al. suggested the diurnal variation of oral glucose tolerance was absent in hyperglycemic individuals [22]. Convincing evidence has shown that the daily rhythms of hepatic glucose production in T2DM contribute to the dawn phenomenon (hyperglycemia in the morning) [26,34]. Additionally, the rhythms in the response of insulin to blood glucose were also dampened in diabetic patients [23]. All in all, there is a great difference in daily glucose metabolism rhythms between healthy humans and diabetic patients [33]. Importantly, T2DM animals and humans also showed circadian disruption in other peripheral tissues and organs. 

#### 3.2.1. Circadian Disruption Occurs in Diabetic Animal Models

In animal models, the T2DM animal-model db/db mice always showed a lower amplitude in locomotor circadian rhythm and body temperature rhythm compared to their controls [35,36]. Disruptive circadian rhythms of blood pressure, baroreflex sensitivity, systolic arterial pressure variance, and sleep-wake patterns were also found in the db/db mice [37,38,39]. In addition, the amplitude or phase in the daily rhythms of the mRNA levels of multiple clock genes, such as *Clock*, *Bmal1*, *Per*, and *Cry*, were partly disrupted in the liver, kidney, adipose tissue, vasculature, and even the submandibular gland [36,37,40,41,42]. Furthermore, a high-fat diet (HFD) could induce obesity and diabetes in mice, which also contributed to disrupted eating behavior, as well as abnormal locomotor activity rhythms [43,44,45,46]. Additionally, the daily rhythms of key clock gene expression levels were disrupted in the liver, kidney, adipose, and hypothalamus of mice under HFD conditions [41,43,44,45,46,47,48]. HFD was even shown to lead to disruption of the rhythms of four core clock genes (*Clock*, *Bmal1*, *Per2*, *Cry1*) in the hippocampus, along with the abnormal rhythms of AD-associated genes and cognition impairment [48]. Streptozotocin (STZ) has been applied in the models of diabetic animals. Studies also found the abnormal circadian rhythms of clock genes dampened in the peripheral tissue and organs like the liver, heart, and gastrointestinal tract in animals receiving STZ injections [49,50,51,52,53]. A lower amplitude of melatonin rhythms was found in the pineal gland, pancreas, kidney and duodenum of rats injected with STZ as compared to controls [54]. The abovementioned animal studies are listed in Table 1.

#### 3.2.2. T2DM Patients Show Disruptive Circadian Rhythms

In studies involving human beings, Lederbogen et al. compared the daily rhythms of blood cortisol levels in 63 ambulatory individuals with T2DM and 916 non-diabetic control subjects, and found a flattened circadian cortisol profile with lower levels in the morning and higher levels in the afternoon and evening in T2DM patients as compared to controls [55]. In addition, other molecules or hormones were also found to display disruptive circadian oscillations in T2DM patients. One study found that the amplitude of daily rhythms in the bone formation marker procollagen type 1 N-terminal propeptide was lower in T2DM compared with the control group, indicating disruption to the circadian rhythms of bone formation [56]. Another study suggested T2DM patients had earlier dim-light melatonin onset, which was regarded as a standard biomarker for estimating circadian phase and higher subjective sleep score (higher score indicated poorer sleep) than the controls [57]. Similarly, the clock genes showed abnormal circadian rhythms and expression levels. For instance, Ushijima found that the clock-associated gene DNA-binding protein (*Dbp*) and peroxisome proliferator-activated receptor γ (*PPAR-γ*) mRNA expression were reduced in omental adipose tissue from donors with gastric cancer and T2DM, compared with those without T2DM [57]. Additionally, several studies reported that the core clock genes and clock-controlled genes (*Clock*, *Bmal1*, *Per*, *Cry*, *Rev-erbα*, *Dpp*) partly exhibited diminished circadian rhythms, which referred to a lower amplitude or shorter period in the islet and leucocytes from T2DM patients [58,59,60,61,62] (Table 2). There are also numerous studies observing sleep disorders in T2DM patients, which may also indicate that the disruption of sleep could modify circadian rhythms [63].

Altogether, these studies suggested that animals and human patients with T2DM were susceptible to disrupted circadian rhythms. The mechanism is largely unknown. Diabetes-associated obesity, hyperphagia, impaired circadian modulation of sympathovagal activity, and inflammation may be involved [36,42,60]. Further research is needed to unravel the exact mechanism.

### 3.3. Circadian Disruption Contributes to T2DM

In addition to the fact that circadian oscillators are damped in T2DM, numerous studies have suggested that circadian disruption contributes to T2DM. Genetic clock-gene disruption in animal models has indicated the critical role of the clock gene in glucose metabolism. For example, Clock mutant mice are obese and hyperphagic, and develop metabolic syndromes including hyperleptinemia, hyperlipidemia and hyperglycemia [64]. The deletion of another key clock gene, Bmal1, in many organs such as the liver, pancreas, and muscles would cause insulin resistance, increased glucose tolerance and hyperglycemia [65,66,67]. Likewise, mice with knockdown of Cry1 and Cry2 in the liver showed increased blood glucose levels, as well as glucagon-stimulated hepatic glucose production [68]. Besides the genetic models, circadian misalignment caused by environmental/behavioral changes also contributed to glucose metabolism disorders in animal and human research [69]. The environmental changes often referred to altered light/dark (LD) cycles, and both the time/period and intensity of light would exert a significant effect on circadian rhythms as the SCN receives light signals [70,71]. Nankivell et al. reported that short photoperiod exposure led to impaired glucose tolerance in *Psammomys obesus* [72]. Constant light exposure caused abolished rhythms in insulin sensitivity in a mouse model [73]. In a prospective cohort study, low-level light at night (LAN) in the bedroom was associated with the increased incidence of diabetes in a general elderly population [74]. The circadian disruption from behavioral changes included disrupted cycles of sleeping/waking, fasting/feeding, rest/activity, and so on. Chronic shift work increased postprandial glucose and decreased insulin sensitivity in healthy individuals and increased the risk of T2DM as well [75,76]. A great number of studies have reported that both sleep deprivation and the mistiming of eating impair glucose tolerance and contribute to the development of diabetes [33,63,69,77]. With respect to how circadian disruption contributed to T2DM, the underlying mechanism may involve an altered sympatho–vagal balance, circadian-regulated hormones, such as glucocorticoid and melatonin, and peripheral clocks that generated tissue-specific rhythmic gene expression to regulate blood glucose [33,69,78]. For example, sleep deprivation led to an impaired sympatho-vagal balance, indicated by changes in heart rate variability [79,80,81], which may contribute to T2DM by decreasing the secretion of insulin, inhibiting insulin-induced glucose uptake and simulating hepatic glucose release [81]. The increased glucocorticoid levels and the inhibition of melatonin under abnormal LD cycles affect blood glucose in various manners, including a decrease in insulin secretion, the exacerbation of insulin resistance and the expression of glucose transporters [78,81,82]. Additionally, the peripheral clocks regulate gluconeogenesis through FOXO1 degradation in the liver and directly affect insulin synthesis as well as secretion in the pancreas. Similarly, the muscle, fat and gut clocks also participate in the regulation of glucose [78]. 

## 4. Circadian Disruption and AD

### 4.1. AD Presents Circadian Disruption

Animal models of AD have been reported to present various kinds of circadian disruptions. Mice overexpressing Amyloid precursor protein (APP) or Aβ showed disruptive circadian rhythms in sleep, locomotor, and body temperature [83,84,85]. Expression of the 0N4R isoform of tau in the clock network of flies led to circadian and sleep defects [86]. Disruptions in circadian rhythmicity in 3xTg-AD mice were even shown to be prior to the expected AD pathology [87]. ApoE^−/−^ mice, which is a model for AD, have been shown to exhibit decreased retinal melanopsin expression, degeneration, and energy shortage in suprachiasmatic and disordered circadian locomotor activity [88]. Such findings in experimental animals also occurred in human studies. Much more severe circadian disruptions, such as higher fragmentations and dampened amplitude, as well as phase shifts, have been observed in patients with AD [89]. Patients with AD showed disrupted daily activity/rest cycles and disruptive cortisol and melatonin rhythms at an early stage [90,91]. An abnormal sleep-wake cycle and melatonin secretion have even become a well-established sign of AD [92,93,94,95]. In addition, AD aggravated the age-associated reduction of the scale invariance of activity fluctuations, reflecting functional changes of the SCN [96], and abnormal clock gene expressions were found in the brain regions of AD patients [97]. To summarize, both animals and humans with AD showed various circadian disruptions.

### 4.2. Effects of Circadian Disruption on AD

That circadian disruption contributes to AD pathology has been reported in many publications [12,98,99]. A shortened 20-h light/dark cycle was reported to contribute to cognitive impairment in mice [100]. A deficiency of several circadian clock genes, including *Clock*, *Bmal1*, *Per*, and *Cry*, has been involved in different AD phenotypes, such as impaired spatial memory, Aβ plaque deposition, and increased astrogliosis [101]. Aβ levels exhibited diurnal fluctuations in both the cerebrospinal fluid (CSF) and interstitial fluid (ISF), the clearance and aggregation of which were regulated by circadian rhythms [99,102]. β-site APP cleaving enzyme 1 (BACE1) and γ-secretase cleave APP to generate Aβ monomers, while A disintegrin and metalloproteinase (ADAM10) plays a protective role by cleaving APP in a non-amyloidogenic manner. It was reported that melatonin could not only decrease Aβ production, through reducing BACE1 and APP expression and increasing ADAM10 expression, but also prevent against tau hyperphosphorylation by inhibiting glycogen synthase kinase-3β (GSK3β) activity and stimulating protein phosphatase-2A (PP-2A) activation [103,104], and its secretion was regulated by circadian rhythms [90]. Besides this finding, studies have suggested that orexin, which is regulated by the sleep-wake cycle, also plays an important role in Aβ dynamics. For instance, sleep restriction led to increased orexin, increasing ISF Aβ levels and brain Aβ plaque deposition [105,106]. Similarly, the ISF and CSF tau, as well as tau pathology-spreading, were also regulated by circadian rhythms, and elevated neuronal metabolism/synaptic strength may enhance tau release and explain increased tau levels under sleep deprivation [107]. Sleep deprivation also accelerated tau pathology, mainly by increasing its more toxic insoluble fraction in AD animal models [108]. Chronic mild sleep restriction was associated with increased cortical Aβ and phosphorylated Tau (pTau) in another publication, in which circulating glucocorticoids may play an important role [109]. Additionally, an altered 6:18 LD cycle was shown to promote AD-associated tau pathology in db/db mice [110]. Moreover, neuroinflammation interfered with the brain’s immunological processes and oxidative stress was associated with the loss of mitochondrial function, both of which could also affect the synaptic activity and aggravate AD-related brain pathology [101,111]. Previous studies suggested that inflammation caused by the activation of glial cells, such as microglia and astrocyte, and gut microbiota were involved in circadian disruption-associated AD progression [12,112]. In addition, cortisol, secreted by the hypothalamic-pituitary-adrenal (HPA) axis, is a well-established circadian rhythm-regulated hormone, which is regulated by the light/dark cycle, sleep/wake cycle, etc. [113]. Activation of the HPA axis was reported to induce cytokines and neuroinflammation, and cortisol could even be used to predict preclinical AD, suggesting that cortisol was closely associated with AD [114,115]. The increased cortisol levels under circadian disruption also contribute to insulin resistance, as well as deficiency in insulin secretion, as referred to in Section 3.3 [82]. The role of cortisol in the relationship between circadian disruption with T2DM and AD seems to be important, while the mechanism underlying circadian misalignment and enhanced oxidative stress possibly lie in dysregulated melatonin and its activation in astrocytes [116,117].

## 5. Treatment for T2DM and AD Targeting Circadian Rhythms

**Figure 2:**
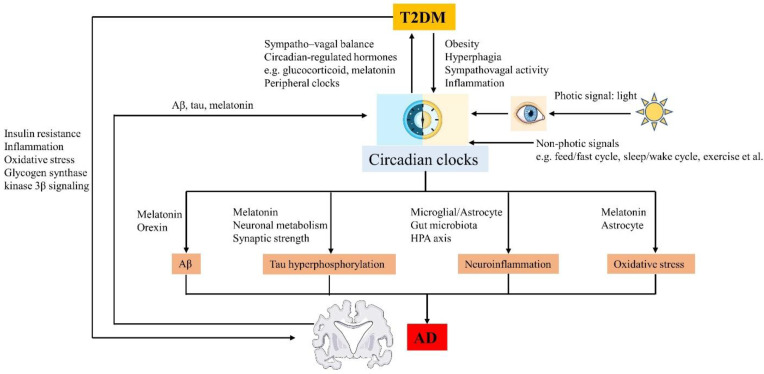
The circadian rhythms, T2DM and AD interact with each other.

**Figure 3:**
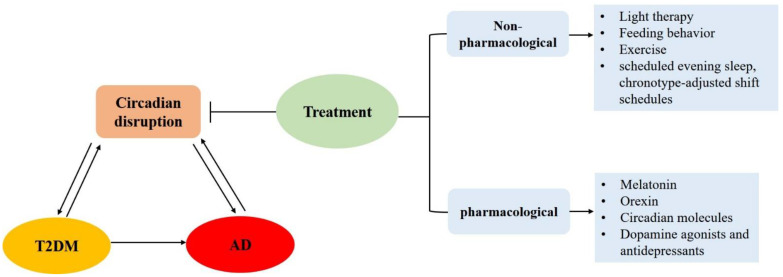
Promising therapeutic strategies targeting circadian disruption for T2DM and AD.

Given that there is much evidence for a close association between circadian disruption, T2DM and AD (Figure 2), targeting circadian dysfunction might provide novel avenues of treatment for both T2DM and AD (Figure 3). Therapeutic strategies targeting circadian rhythms for T2DM and AD are discussed in the following section. The relevant treatment targeting circadian rhythms in human studies are summarized in Table 3.

### 5.1. Non-Pharmacological Treatment

#### 5.1.1. Light Therapy

The SCN mainly receives light signals, so the optimization of daily light exposure can be used to increase circadian synchrony [118]. Bright light in the morning (bright light therapy, BLT) has been used as a treatment for mood disorders, depression and circadian abnormalities, including shift work and sleep disorders [119,120]. High-intensity light exposure daily in the morning contributed to the proper function of the circadian system and, therefore, lowered body weight and improved glucose tolerance in sand rats [121]. A randomized, double-blind trial suggested BLT may be promising in the treatment of depression among T2DM patients with high insulin resistance [122]. In addition, Roccaro et al. performed a literature search about BLT on sleep-wake patterns in AD over the latest 20 years and found that using light as a non-pharmacological treatment was able to improve circadian rhythms in AD patients [122,123]. Therefore, stimulating the SCN by BLT may be of great value in the treatment of circadian-related T2DM, as well as that of AD patients.

#### 5.1.2. Feeding Behavior

Growing evidence suggests that feeding behavior could also regulate circadian clocks [124,125]. The chrono-nutrition pattern for T2DM is generally as follows: calorie restriction (CR), characterized by a reduced average daily caloric intake, intermittent fasting (IF), where you cycle between periods of eating and fasting, and time-restricted feeding (TRF), in which food is restricted in a certain period [126]. The benefits made by these three feeding behaviors have been well established in T2DM treatment [126,127,128]. At least some beneficial outcomes of CR in the metabolism are due to its effect on the circadian clock [129]. Likewise, IF and TRF also improve metabolic disorders by restoring a healthier circadian clock, in which the gut microbiota is involved as well [130]. For their effects on AD, although there are numerous studies suggesting the protective effect of CR and IF against AD, the role of the circadian clock was seldom reported in the research [131,132,133,134]. TRF was shown to improve circadian dysfunction and motor symptoms, in the mouse model of Huntington’s Disease [135]. TRF could also improve cognitive function in older adults [136], although the mechanism was uncertain. As AD is often accompanied by circadian disruption, the CR, IF and TRF might be promising interventions against T2DM and AD from the perspectives of the circadian clock. Future research should look into the role of circadian rhythms in terms of the effect of feeding behaviors on AD.

#### 5.1.3. Exercise

Besides the LD cycle and feed/fast cycle, exercise as an external environmental cue could also serve as a potent entrainment signal for circadian clocks [137]. Exercise could shift the phase of circadian rhythms, including wheel-running behavior and the sleep-wake schedule, as well as melatonin, which brings many benefits in treating circadian disruption [138,139,140]. Exercise training improves insulin resistance and decreases HbA1c, which therefore reduces the risk of diabetic complications [141,142]. A recent study indicated that a 12-week exercise training regime increased skeletal muscle BMAL1 gene expression and PER2 protein expression in adults with obesity and prediabetes, which was associated with enhanced peripheral insulin sensitivity [143]. It is tempting to speculate that the beneficial metabolic effects of exercise training may be in part mediated by the circadian timing system [78,143]. Exercise could also decrease Aβ accumulation, the phosphorylation of tau, inflammation and the synthesis and release of neurotrophins, thus protecting against AD [144]. Some studies have suggested that the positive benefits of exercise for improving cognition could be mediated through a diurnal cycle of cortisol secretion [145,146]. Another study found that the combination of BLT with restricted periods of exercise improved circadian rhythmicity in the case of neurodegenerative Huntington’s disease [147]. Thus, it is possible that exercise could also improve AD through circadian rhythms. Exercise training targeting circadian rhythms might be an alternative non-pharmacological intervention for T2DM and AD.

#### 5.1.4. Other Lifestyle Interventions

Some interventions, such as scheduled evening sleep, improved sleep quality and enhanced social interactions could help develop normal, healthy circadian rhythms [78,148,149]. In addition, chronotype-adjusted shift schedules were supposed to align work and circadian time, consequently reducing the circadian disruption in shift workers [150]. Other recommendations, such as avoiding overlong work hours, prolonging shift intervals, and reducing the shift duration and the number of consecutive night shifts could be applied in the improvement of circadian rhythms in shift work [148,151]. These lifestyle interventions may also be effective ways of improving circadian rhythms and delaying both T2DM and AD.

### 5.2. Pharmacological Treatment

#### 5.2.1. Melatonin

In modern society, circadian disruptions, such as excessive artificial light at night, shift and/or night work and jet lag, contribute to disrupting the rhythms of melatonin and suppressed secretion; they also trigger sleep deprivation and the onset of diseases like T2DM and AD [98,152,153,154,155]. Melatonin is a hormone produced in the pineal gland, the production of which is tightly controlled by the SCN, increasing at night and decreasing during the daytime. There are many benefits associated with melatonin. Firstly, melatonin can serve as an entrainment signal for the circadian system [156]; melatonin has established sleep-promoting effects, which means that it may serve as a therapeutic agent for treating sleep and circadian rhythm disorders [152,155,157,158]. Secondly, there is evidence indicating that melatonin could improve glucose homeostasis and insulin resistance in rodent animals [155]. Several studies also showed the protective role of melatonin in sleep quality and blood glucose control in T2DM patients [159,160]. In addition, melatonin treatment reduced Aβ accumulation, tau hyperphosphorylation and oxidative stress, and improved impaired cognition in AD animal models [157]. Patients with AD who were additionally treated with melatonin showed better cognitive performance than those treated with a placebo in a 6-month multicenter clinical trial [161]. A double-blind study of melatonin in AD demonstrated decreased nocturnal activity, increased nocturnal sleep, and cognition improvement [162]. Moreover, one recent study showed that melatonin could prevent cognitive dysfunction in T2DM mice [163], while another recent study indicated that a lower melatonin level was related to cognitive impairment in T2DM patients [164]. Therefore, melatonin might be used as a potential protective molecule against both T2DM and AD. Given that the current relevant research is limited, further investigations are needed to verify its effect against AD and T2DM.

#### 5.2.2. Orexin

Orexin, including orexin A and orexin B, is a neuropeptide hormone synthesized in the lateral hypothalamus (LH), which plays a significant role in circadian rhythms such as sleep homeostasis and feeding behavior [165,166]. Orexin, promoting wakefulness, was thought to modulate glucose metabolism by connecting its clock and glucose rhythmicity [167]. In T2DM mice, orexin antagonists provided chronotherapeutic effects against disturbances and improved glucose intolerance [168,169]. In patients with T2DM and insomnia, a selective orexin receptor antagonist not only improved sleep disorders but also provided metabolic benefits like abdominal circumference [170]. While controversial studies exist, some studies found that diabetic mice showed suppressed orexin expression [171,172], and orexin administration may have beneficial effects. One previous study confirmed that orexin could prevent hepatic insulin resistance via regulating daily blood glucose oscillation in T2DM mice [173], while another randomized clinical trial found an inverse association of peripheral orexin-a with insulin resistance in T2DM patients [174]. Hence, treatment targeting orexin may depend on sleep-states in T2DM. In terms of the role of orexin in AD, as orexin participated in the modulation of circadian oscillations in the levels of Aβ and AD and often showed correlation with sleep disorders, the benefits of orexin antagonists in AD have been reported in some research [99,175]. Ma et al. found orexin-signaling regulated the hippocampal clock and the circadian oscillation of AD-risk genes [176]. Orexin was considered to exacerbate Aβ accumulation in AD mice [177], and an orexin antagonist could improve circadian rhythms, reduce the Aβ plaque burden and improve AD in animals and humans [178,179,180]. Thus, orexin is also a promising drug against T2DM and AD by targeting circadian rhythms.

#### 5.2.3. Circadian Molecules

New circadian therapies are looking for clock-improving molecules from large-scale chemical screens [78]. It was reported that REV-ERB agonists, such as SR9011, SR9009, ROR agonists and CRY stabilizers, which directly targeted the molecular clock, were promising candidates for improving obesity as well as glucose metabolism in T2DM animal models [78,181]. Nobiletin, a natural polymethoxylated flavone, could be used as a clock amplitude-enhancing small molecule [78,182]. A previous study suggested that nobiletin counteracted metabolic syndrome and improved locomotor activity in a clock gene-dependent manner in mice models, indicating its effect in enhancing circadian rhythms to combat metabolic disease [182]. Some studies involving circadian molecules are also found in AD models. Roby et al. reported that using SR9009 to pharmacological activate the nuclear receptor REV-ERB reduced Aβ levels and reversed cognitive deficits in an AD mice model [183]. Guo et al. showed that the application of the REV-ERBα agonist GSK4112 or SR9011 dose-dependently inhibited LPS-induced microglial activation and could be applied in protecting neurons from neuroinflammation [184]. In addition, a recent publication showed the clock modulator nobiletin was able to activate circadian cellular oscillators and strongly ameliorated Aβ pathology in female mice in an AD model [185]. Collectively, these studies demonstrated that the circadian clock is a modifiable target for treating T2DM and AD. However, there are no studies exploring the role of these molecules in T2DM or AD in human research; further studies are expected to reveal their promising effect.

#### 5.2.4. Other Drugs

It is reported that dopamine agonists and a low dose of antidepressants could improve sleep disorders in T2DM [63]. Dopamine could modulate the circadian rhythm and associated metabolic changes, which might serve as a therapeutic agent for diabetes [186]. Bromocriptine, as a dopamine D(2) receptor agonist, could improve insulin resistance and other metabolic dysfunction through regulating the circadian rhythm, and has been used for the treatment of T2DM in the United States [187,188]. Dopamine is thought to improve sleep quality, and low-dose risperidone administration increased the binding potential of the dopamine receptor and improved both the sleep/wake patterns and behavioral abnormality via blocking the serotonin system in AD patients [189]. In addition, the dopamine D1 receptor agonist improved Aβ_1-42_-induced cognitive dysfunction and inflammation [190]. Moreover, antidepressants could also improve mood disorders and sleep quality and have favorable effects on glycemic control in T2DM [191]. A recent study reported that circadian disruption by chronic constant light led to AD progression, while fluoxetine prevented this effect [192]. Another study showed that the antidepressant agomelatine could rescue streptozotocin-induced AD pathology, including Aβ accumulation and neuroinflammation [193]. Last but not least, studies have reported that serum YKL-40, an inflammatory cytokine, increased in diabetic patients and is associated with the increasing severity of albuminuria. A recent study found plasma YKL-40 is upregulated in T2DM-associated cognitive impairment, compared to those with normal cognition in T2DM patients [194]. The YKL-40 was also regulated by the circadian clock; as one study suggested, CSF YKL-40 showed a diurnal variation, and its absence could be used as a candidate marker of AD [195]. Another recent study even reported that the astrocyte circadian clock regulated inflammatory YKL-40, the increased expression of which promoted Aβ plaque in AD mice and humans [196]. Thus, the inhibition of YKL-40 through the circadian clock might be a prospective therapeutic target for slowing the progression of AD in T2DM.

Therefore, dopamine agonists, antidepressants, and drugs targeting YKL-40 might also be promising candidates for circadian disruption-induced T2DM and AD, but further research is needed in both humans and animals.

## 6. Conclusions

T2DM is associated with an increased risk of developing AD [197]. In recent years, circadian rhythms, T2DM and AD have been identified as interacting with each other [9]. It is apparent that considerable circadian disruption occurs in T2DM animals and patients, including the abnormal secretion of circadian-regulated hormones, sleep disorders, abnormal clock gene expression in peripheral tissue, and so on. The circadian rhythm system has been increasingly found to play an important role in AD [95,99]. Circadian disruptions, such as an altered LD cycle, an abnormal sleep-wake cycle, and shift work contribute to the progression of AD [98,198]. Considering the vital role of circadian rhythms, treatment targeting circadian rhythms might be a promising intervention for T2DM and AD. Possible pharmacological treatment, such as melatonin, orexin, circadian molecules, as well as non-pharmacological treatments like light therapy, feeding behavior modification, exercise and other lifestyle interventions, may be attractive candidates for treating T2DM and AD from the circadian perspective. We expect further investigations of the effect and mechanism of these promising treatments in the coming years.

## Figures and Tables

**Table 1:** Circadian rhythm disruptions among diabetic animal models.

First Author, Year	Animals	Age	Type of Circadian Markers	Results
Hou, 2019	Db/db, Db/+ mice	16–24 w	Daily rhythms of BP, baroreflex sensitivity and mPer2^Luc^ oscillations	Db/db mice had disrupted daily rhythms of BP, baroreflex sensitivity, and advanced phase shift of mPer2 daily oscillation in the liver, kidney, and submandibular gland.
Grosbellet, 2016	Db/db, Db/+ mice	10 w	Body temperature rhythm, general activity rhythm	Db/db mice had lower amplitude in body temperature rhythm and general activity rhythm under normal LD cycle and had a longer endogenous period for both activity and temperature rhythms compared with db/+ mice under constant darkness.
Su, 2012	Db/db, Db/+ mice	9–10 w	Daily rhythms of mRNA levels of multiple clock genes (*Clock*, *Bmal1*, *Per1/2*, *Cry1/2*, *Rev-Erba*) and target genes (*Dbp* and *Pparγ*) in the aorta, mesenteric arteries, heart, kidney, and SCN	Db/db mice had suppressed 24-h mRNA rhythms of the following clock and target genes: *Per1/2*, *Cry1/2*, their target genes, *Dbp* and *Pparγ*, in the aorta and mesenteric arteries; *Dbp* in the heart; *Per1*, *Rev-Erba*, and *Dbp* in the kidney; *Per1* in the SCN.
Caton, 2011	Db/db, Db/+ mice	8 w	Expression levels of Clock, Bmal1, Per2, Cry1 in mRNA and protein in WAT	Db/db mice had lower expression of *Clock* mRNA (42%), CLOCK protein (72%), BMAL1 protein (34%; but not mRNA), *Per2* mRNA (17%) in WAT, while no difference was found in Cry1 in WAT, compared with db/+ mice.
Senador, 2009	Db/db, Db/+ mice	7–8 w	Circadian rhythms of systolic arterial pressure variance and its low-frequency component	Circadian rhythms of systolic arterial pressure variance and its low-frequency component are absent in db/db mice
Su, 2008	Db/db, Db/+ mice	15–17 w	Oscillations of clock genes *DBP* and *Bmal1* in vasculature; circadian rhythms of BP, heart rate, and locomotor circadian rhythm	Db/db mice had a disrupted BP, heart rate, and locomotor circadian rhythm associated with dampened oscillations of clock genes *Dbp* and *Bmal1* mRNA in vasculature.
Laposky, 2008	Db/db, wt/wt mice	14–18 w	Diurnal rhythmicity of sleep-wake patterns	Db/db mice had increased total sleep time, sleep fragmentation and attenuated the daily rhythm of the sleep-wake cycle.
Kudo, 2004	Db/db, Db/+ mice	13–14 w	Daily oscillations of *Per2* and *Bmal1* mRNA expression in liver	The expression of *Per2* mRNA levels was severely diminished and the phase of *Bmal1* mRNA oscillation was advanced in the db/db mouse liver.
Woodie, 2020	C5Bl/6N	7 w; HFD 16 w	Daily rhythms of mRNA expressions of clock genes (*Clock*, *Bmal1*, *Per2*, *Cry1*) in the liver, hypothalamus and hippocampus	HFD caused disruptive mRNA expression rhythms of *Bmal1* in the liver, *Cry1* in the hypothalamus and all core clock genes (*Clock*, *Bmal1*, *Per2*, *Cry1*) in the hippocampus along with abnormal rhythms of AD-associated genes.
Katrina, 2015	C57BL/6J	8 w; HFD 5 w	Eating behavior and locomotor activity rhythms; PER2:LUC bioluminescence rhythms in liver	HFD disrupted eating behavior and locomotor activity rhythms; The phase of Per2 was advanced by 4 h in the liver.
Pendergast, 2013	C57BL/6J	7 w; HFD 1 w	PER2 expression in the gonadal white adipose tissue (surrounding the gonads), liver, lung, spleen, aorta, pituitary, SCN and arcuate complex	the phase of the PER2 rhythm was markedly advanced (by 5 h) in the liver of HFD mice, whereas rhythms in other tissues were not affected.
Hatori, 2012	C57BL/6J	12 w; HFD 6 w	Diurnal rhythms in food intake and RER; the oscillations of circadian clock genes (*Per1*, *Per2*, *Cry1*, *Bmal1*, *Clock*, *Rorα Rev-erbα*, *Dbp*) mRNA levels	HFD damped diurnal rhythms in food intake and RER in mice; HFD also dampened the oscillations of circadian clock components (*Per1*, *Per2*, *Cry1*, *Bmal1*, *Clock*, *Rorα Rev-erbα* and *Dbp*) in the liver.
Caton, 2011	C56Bl/6	8 w; HFD 16 w	Expression levels of clock, bmal1, per2, CRY1 in mRNA and protein in WAT	Clock mRNA (60%) and protein levels (42%) were decreased in WAT of HFD mice compared to control.
Hsieh, 2010	C57BL/6	HFD 11 m	The mRNA expressions of circadian-clock genes and clock-controlled genes, including *Per1-3*, *Cry1-2*, *Bmal1*, *Dbp*, *E4BP4*, *CK1varepsilon*, *PEPCK*, *PDK4* and *NHE3* in the liver and kidneys	HFD disrupted the circadian rhythms of *Per1-3*, *Cry1-2*, *Bmal1*, *Dbp*, *E4BP4*, *CK1varepsilon*, *PEPCK*, *PDK4* and *NHE3* in the liver and kidneys.
Kohsaka, 2007	C57BL/6J	6 w; HFD 6 w	Free-running period, feeding behavior rhythms, Clock, Bmal1 and Per2 m RNA expression in the fat and liver.	HFD lengthened the free-running period in mice and attenuated the diurnal pattern of feeding behavior. The amplitudes of *Clock*, *Bmal1* and *Per2* mRNA expression were decreased in both the fat and liver of the HFD mice.
Yang, 2013	C57BL/6	Postnatalday 2 with a single injection of STZ	Circadian clock genes mRNA levels in the livers of mice at the age of 16 weeks	Only *Bmal1*, *Cry1* and *Per2* mRNA expressions were elevated for the group injected with STZ on the postnatal day 2.
Bostwick, 2010	C57BL/6J	10–12 w; STZ injection once a day for 4 days	The mRNA expressions of *Per* genes in the stomach body, proximal and distal colon, liver, kidney and lung	*Per2* and *Per3* mRNA expression levels of STZ-injected mice were generally phase-delayed within the gastrointestinal tract but not within the kidney or lung in acute (1 week) and chronic (12 weeks) STZ-induced diabetes compared with control mice, although the rhythmicity in expression of *Per2* and *Per3* persisted in all organs.
Stebelová, 2007	Wistar rats	One single injection of STZ	Daily rhythm of melatonin concentrations in the pineal gland, plasma, pancreas, kidney, spleen and duodenum	The diabetic group resulted in lower melatonin levels in the pineal gland, pancreas, kidney and duodenum as compared to control, while no difference was found in the spleen on day 17 after STZ treatment.
Herichová, 2005	Wistar rats	8 w; STZ injection once	The mRNA expression of *Per2* and *Dbp* in the heart and liver.	The rhythm in per2 and dbp expression was slightly advanced in hearts, although the rhythms existed; the daily rhythm of *Per2* was lost and *Dbp* showed a similar advanced shift in the liver of mice 4 weeks after the STZ injection.
Kuriyama, 2004	ddY mice	15 w with a single injection of STZ	Rhythmic expression of *Per2* mRNA and protein in the SCN, cerebral cortex and liver	mRNA and protein expression levels of *Per2* were dampened in the liver but not SCN and cerebral cortex of mice on the fifth day after injection with STZ.
Young, 2002	Wistar rats	One single injection of STZ	Daily rhythms of mRNA expressions of clock genes (*Bmal1*, *Clock*, *Cry*, *Per)*, as well as three clock output genes (*Dbp*, *Hlf* and *Tef*) in the hearts	A phase shift (approximately 3 h early) was observed for the mRNA expression levels of *Bmal1*, *Per*, *Cry* and the three output genes (*Dbp*, *Hlf* and *Tef*) in the hearts of mice in 4 weeks after the initial STZ injection.

Clock: circadian locomotor output cycles kaput; Bmal1: brain and muscle arnt-like protein-1; Dbp: DNA-binding protein; Per: period; Cry: cryptochrome; PPAR-γ: peroxisome proliferator-activated receptor γ; SCN: suprachiasmatic nucleus; RER: respiratory exchange ratio; WAT: white adipose tissue; HFD: high-fed diet; STZ: streptozotocin; Hlf: hepatic leukemia factor; TEF: thyrotroph embryonic factor.

**Table 2:** Circadian rhythm disruptions among T2DM patients.

First Author, Year	Participants	Type of Circadian Markers	Results
Ushijima, 2020	13 non-T2DM and 12 T2DM with gastric cancer	Clock associated gene *Dbp* and *PPAR-γ* mRNA expression in omental adipose tissue	*DBP* and *PPAR-γ* mRNA expression are reduced in omental adipose tissue in T2DM patients.
Petrenko, 2020	12 T2DM patients and 27 nondiabetic patients	Clock genes *(Clock*, *Bmal1*, *Per*, *Cry*, *Rev-erbα*, *Dpp*) mRNA expression levels in human islets from T2DM and nondiabetic donors	mRNA expression levels of *Per*1-3, *Cry*2, *Rev-erbα*, *Clock and Dbp* were significantly diminished in T2D compared to nondiabetic islet cells combined with, while BMAL1 and CRY1 did not change.
Ando, 2020	Study 1: 8 T2DM patients and 6 comparatively young non-diabetic volunteers Study 2: 12 male T2DM patients and 14 age-matched men	Clock genes (*Clock*, *Bmal1*, *Per1*, *Per2*, *Per3* and *Cry1*) mRNA expression levels at 9 a.m., 3 p.m., 9 p.m., and 3 a.m. (study 1) and at 9 a.m. (study 2) in peripheral leucocytes	In study 1, mRNA expression levels of *Bmal1*, *Per1*, *Per2* and *Per3* were significantly lower in T2DM patients than in non-diabetic individuals at one or more time points. In study 2, lower transcript levels of *Bmal1*, *Per1* and *Per3* were found in leucocytes obtained from T2DM patients than in control individuals, and the transcript expression was inversely correlated with HbA(1c) levels.
Yu, 2019	36 T2DM patients and 14 non-diabetic volunteers	Transcript levels of circadian clock genes (*Clock*, *Bmal1*, *Per1*, *Cry1* and *Cry2*) in peripheral blood leucocytes	The T2DM patients had lower CLOCK, BMAL1, PER1, CRY1 and CRY2 mRNA levels than nondiabetic participants in peripheral blood leucocytes. Blood inflammatory markers (IL-6, TNF-α) HbA1c levels were negatively correlated with *Bmal1*, *Per1* and *Cry1* mRNA levels.
Hygum, 2019	5 T1DM, 5 T2DM patients and 5 controls (age > 50 years)	24-h variation of bone formation	The rhythms of bone formation marker procollagen type 1 N-terminal propeptide were lower in T2DM compared with controls.
Sinturel, 2019	9 obese and 8 non-obese individuals with T2DM and 11 non-diabetic controls	Rhythms of clock gene Bmal1 in dermal fibroblasts established from skin biopsies	The oscillation period of the *Bmal1-luc* reporter was significantly shorter in the type 2 diabetes group (particularly the obese subgroup) than controls. HbA1c values were found to be significantly inverse (ρ = −0.592; *p* < 0.05) with the circadian period length within cells from the T2DM group
Dumpala, 2019	23 patients with T2DM and 24 age-matched healthy controls	DLMO; sleep questionnaires; light exposure measured by actigraphy	T2DM had earlier DLMO (1 h), higher subjective sleep score than controls although no significant difference was found in light exposure pattern.
Perciaccante, 2016	90 Caucasian IR subjects (divided into four groups: IR with normal OGTT results, IR with IFT, IR with IGT and T2DM) and 25 control subjects	Autonomic nervous activity measured by 24-h ECG recording and heart rate variability	The IR groups all showed impaired autonomic activity reflected by sympathovagal balance (expressed by the LF/HF ratio) and reduced standard deviation of all sinus rhythm RR values compared to the controls.
Stamenkovic, 2012	5 T2DM patients and 55 nondiabetic controls	The core clock genes (*Clock*, *Bmal1*, *Per1* to *3*, *Cry1* and *Cry2*) in islets from donors	The mRNA levels of *Per2*, *Per3* and *Cry2* were significantly lower in islets from donors with T2DM than the non-diabetic controls. mRNA levels of *Per2*, *Per3*, and *Cry2* correlated positively with insulin content, and the expression of *Per3* and *Cry2* correlated negatively with glycated hemoglobin levels.
Lederbogen, 2011	63 ambulatory individuals with T2DM and 916 non-diabetic control subjects	Saliva cortisol concentrations on waking, a salivette ½ h, 8 h and 14 h after waking	Diabetic subjects had a flattened circadian cortisol profile, with lower levels in the morning and higher levels in the afternoon and evening.

Clock: circadian locomotor output cycles kaput; Bmal1: brain and muscle arnt-like protein-1; Dbp: DNA-binding protein; Per: period; Cry: cryptochrome; PPAR-γ: peroxisome proliferator-activated receptor γ; IL-6: Interleukin 6; TNFα: tumor necrosis factor α; IR: insulin-resistant; IFG: impaired fasting glucose; IGT: impaired glucose tolerance; DLMO: dim light melatonin onset; LF/HF: low frequency/high frequency.

**Table 3:** Therapeutic strategies targeting circadian rhythms for T2DM and AD in human studies.

First Author, Year	Study Design	Participant	Treatment	Type of Circadian Markers	Results
Brouwer, 2015	RCT	83 adult T2DM patients with major depressive episodes (mean age = 60.1/62.9 years)	Light therapy (10,000 lux) for 30 min every morning for 4 weeks at home	Sleep (duration, efficiency, time)	Light therapy did not result in significant changes in sleep duration, sleep efficiency, or mid-sleep time. Light therapy did not affect depressive symptoms in participants with higher insulin sensitivity, but it did produce an anti-depression effect in participants with lower insulin sensitivity.
Yamadera, 2000	RCT	27 adults with AD (mean age = 79.9 years)	Light therapy (3000 Lux; 9–11 a.m.) for 4 weeks	Sleep/nap time, awakenings in the night	The therapy improved circadian rhythm disturbances and MMSE scores, especially in the early stages of AD, although the CDR scores were not improved.
Gabel, 2019	RCT	43 insulin-resistant subjects (mean age = 44 years)	IF (25% of energy needs on “fast days”; 125% of needs on alternating “feast days”) or CR (75% of energy needs every day) for 12-month	None	IF and CR caused similar decreases in body weight compared with the control group. IF contributed to greater reductions in fasting insulin and insulin resistance than CR.
Parr, 2020	RCT	11 sedentary males (mean age = 38 years; mean BMI = 32.2 kg/m^2^)	Two isoenergetic diet protocols for 5 days, consuming meals at 10 a.m., 1 p.m. and 5 p.m. (TRF) or 7 a.m., 2 p.m., and 9 p.m. (EXF).	None	Total 24-h area under the curve of glucose tended to be lower for TRF versus EXF (−5.5 ± 9.0 mmol/L/h, *P* = 0.09). Area under the curve of nocturnal glucose was lower in TRF (−4.2 ± 5.8 mmol/L/h, *P* = 0.04).
Currenti, 2021	observational study	883 adults (age ≥ 50 years). Participants with an eating time window of less than 10 h over the last 6 months were identified as the TRF group.	None	None	Individuals adhering to TRF were less likely to have a cognitive impairment, compared to those with no eating time restrictions (OR = 0.28; 95% CI: 0.07–0.90).
Erickson, 2020	RCT	24 adults with obesity and prediabetes (mean age = 66 mean BMI = 34 kg/m^2^ mean fasting plasma glucose = 105 mg/dL)	Exercise intervention for 12 weeks (5 days per week at ~85% of heart rate max on a treadmill for 60 min)	Expression of circadian clock genes (BMAL1, CLOCK, CRY1/2, and PER 1/2) in skeletal muscle	BMI, peripheral insulin sensitivity and exercise capacity all improved (*P* < 0.005) with exercise training. Skeletal muscle *BMAL1* gene (fold change, 1.62 ± 1.01; *P* = 0.027) and PER2 protein expression (fold change, 1.35 ± 0.05; *P* = 0.02) increased, whereas CLOCK, CRY1/2, and PER1 were unchanged. The fold change in BMAL1 correlated with insulin sensitivity (r = 0.43, *P* = 0.044), BMI (r = −0.44, *P* = 0.042), and body weight changes (r = −0.44, *P* = 0.039).
Dijckmans, 2017	observational study	cognitive impairment group (*n* = 30) and normal group(*n* = 30) (mean age = 70.6)	None	Cortisol circadian rhythms	Better cognitive function was associated with better physical performance. A greater variance in cortisol levels across the day from morning to evening was associated with better cognitive function and physical performance.
Garfinkel, 2011	RCT	36 independently living T2DM patients with insomnia (mean age = 63)	Period 1: treatment with prolonged-release melatonin (2 mg) or placebo for 3 weeks Period 2: treatment for another 3 weeks after a one-week washout periodPeriod 3: treatment for an extension period of 5 months	Sleep efficiency, wake time after sleep onset, and number of awakenings	3 weeks of prolonged-release melatonin treatment improved sleep quality. Following 5 months of prolonged-release melatonin treatment, HbA1c was significantly lower than at baseline (9.13% ± 1.55% versus 8.47% ± 1.67%, respectively, *P* = 0.005).
Asayama, 2003	RCT	20 AD patients divided into placebo group (*n* = 9) and melatonin group (*n* = 11) (mean age = 79.2 years)	Melatonin (3 mg) were given at 8.30 p.m. each day for 4 weeks	sleep time and activity	Melatonin administration had the effect of improving sleep time, night activity and ADAS scores
Zarifkar, 2017	RCT	59 newly diagnosed T2DM patients (30 in the metformin group and 29 in the pioglitazone group	Treatment with either metformin (1000 mg daily) or pioglitazone (30 mg daily) for 3 months	orexin	Three-month anti-hyperglycemic treatment with proportionate doses of metformin or pioglitazone both improve insulin resistance and glycemic control. A negative association between peripheral orexin concentrations and insulin resistance was observed in T2DM patients.
Herring, 2020	RCT	285 participants with AD and insomnia (suvorexant, N = 142; placebo, N = 141, mean age = 69)	Treatment with 10 mg suvorexant (an orexin antagonist) (could be increased to 20 mg based on clinical response) or a placebo for 4 weeks	total sleep time	Suvorexant improved total sleep time in patients with probable AD dementia and insomnia.

RCT: randomized controlled trial; MMSE: mini-mental state examination; CDR: clinical dementia rating; IF: intermittent feeding; CR: caloric restriction; BMI: body mass index; TRF: time-restricted feeding; EXF: extended feeding; OR: odds ratio; CI: confidence intervals: ADAS: Alzheimer’s Disease assessment scale.

## Data Availability

No new data were created or analyzed in this study. Data sharing is not applicable to this article.
